# SEC61G Promotes Cervical Cancer Proliferation by Activating MAPK Signaling Pathway

**DOI:** 10.1155/2022/7016079

**Published:** 2022-08-31

**Authors:** Yangyang Fan, Ying Wang, Feifei Liu, Haili Wang, Qiumin Li

**Affiliations:** Department of Obstetrics and Gynecology, Shaanxi Province People's Hospital, Xi'an, 710068 Shaanxi, China

## Abstract

**Objective:**

The abnormal expression of SEC61G plays an important role in the development of various tumors. This study explored the effects of SEC61G on MAPK signaling pathway and proliferation of cervical cancer (CC) cells.

**Methods:**

shRNA was used to inhibit the expression of SEC61G and EdU to observe its effect on the proliferation of CC cell SiHa. The effect of SEC61G on invasion was evaluated by Transwell assay. TCGA database was used to analyze the influence of high or low SEC61G expression level on the overall survival of CC patients. Western blot was used to detect the expressions of SEC61G, p-RAF1, Raf1, p-MEK1/2, MEK1/2, and p-ERK1/2 in cells. SiHa cells overexpressing SEC61G (SiHa-SEC61G) and control group (SiHa-mock) were subcutaneously implanted in nude mice. The tumor growth curve was measured at the specified time points between SiHa-SEC61G and SiHa-mock. The inhibitory effect of gefitinib on SEC61G was further evaluated.

**Results:**

In patients with CC, high SEC61G expression predicted poor prognosis. Silencing SEC61G inhibited proliferation and invasion of CC cells in vitro. Overexpression of SEC61G can promote the proliferation and invasion of CC cells in vitro. Meanwhile, overexpression of SEC61G promoted the proliferation of CC xenografts. Knocking down SEC61G can inhibit MAPK signaling pathway. Gefitinib can inhibit CC proliferation and tumor growth by SEC61G.

**Conclusion:**

SEC61G is highly expressed in CC and has poor prognosis. Inhibition of SEC61G expression can effectively inhibit the growth and proliferation of human CC cells. The mechanism may be related to the inhibition of MAPK signaling pathway.

## 1. Introduction

Cervical cancer is not easy to be diagnosed early because of its hidden location, and it is easy to relapse and metastasis [1]. Although there are many researches on its pathogenesis, the overall clinical therapeutic effect is still not ideal [2]. Finding molecular markers for early diagnosis, therapeutic targets, and prognostic monitoring factors plays an important role in the treatment of CC [3, 4].

Sec is a key signal pathway regulating protein transport, which exists widely in all types of cells [5]. The key protein controlling the Sec system in eukaryotes is the Sec61 complex [6]. Sec61 channel proteins constitute a generally conserved protein transduction channel that transports and integrates proteins into the eukaryotic endoplasmic reticulum (ER) membrane and the plasma membrane of prokaryotic cells [7]. In glioblastoma, SEC61G is used as a new prognostic marker to predict patient survival and treatment response [8]. As a channel protein, the role of Sec61G in lung cancer is unclear.

Mitogen-activated protein kinase (MAPK) signal transduction pathway is closely related to tumorigenesis [9]. MAPK signaling pathway is a complex network system, which plays an important role in the process of cell proliferation, apoptosis, invasion, metastasis, and vascular formation [10]. The biological function of MAPK signal transduction pathway is realized through continuous phosphorylation of MAPKKK (mitogen-activated protein kinase kinase kinase kinase), MAPKK (mitogen-activated protein kinase kinase kinase), and MAPK [11]. After MAPK is activated, translocation into the nucleus can phosphorylate of some transcription factors in the nucleus [12]. Phosphorylation initiates transcription and translation of primary and secondary response genes and regulates the activity of some protein kinases. Inactivation of MAPK is caused by the phosphorylation of threonine and tyrosine by a group of bispectral protein kinases (MKPs), returning it to its ground state [13].

In this study, RNA interference was used to inhibit the expression of SEC61G gene and observe its effect on proliferation of CC cells. The possible mechanism of MAPK signaling pathway was discussed to provide experimental basis for elucidating the pathogenesis and treatment of CC.

## 2. Methods

### 2.1. Survival Analysis

It was through The Human Protein Atlas database (https://www.proteinatlas.org/) analysis of high or low expression level SEC61G queue overall survival in patients with cervical cancer (CC) of Kaplan-Meier curve.

### 2.2. Cell Culture

Human cervical epithelial cells (CerEpiC) and human cervical cancer cell lines HeLa, C33A, and SiHa and CaSki were cultured in PRMI 1640 medium containing 10% fetal bovine serum and in 37°C, 5% CO_2_ incubator. The cell growth was observed under an inverted microscope. The medium was replaced every 2 days, and the cells grew to about 80% and were digested with trypsin for passage. Cells in good growth condition were taken for experiment.

### 2.3. Cell Transfection

Cells in good growth condition were taken, and 6-well plates were laid on the first 1 day night. The cell density of each 6-well plate was about 70%~80%. Make sure the cells are evenly spread on the bottom of the plate. Plasmid transfection was performed shortly after cell adherence in the morning of day; 2.5 *μ*g plasmid and 10 *μ*L Lipo3000 were diluted in 125 *μ*L OPTI-MEN. In addition, 3.75 *μ*L Lipo3000 was also diluted in 125 *μ*L OPTI-MEN. Then, mix the two and let stand at room temperature for 5 min. Add to 6-well plate with 2.25 mL residual medium. After 5 h, the solution was changed for subsequent experiments.

### 2.4. EdU Experiment

Cells 24 h after transient transfection were placed on 96-well plates. The cells were resuspended into single-cell suspension with 5 × 10^3^ cells per well and 100 *μ*L culture base. Each group has 3 compound holes. The cells were cultured in a 5% CO_2_ incubator at 37°C. EdU detection was after 12 h. Cells were cultured with 1000 : 1 diluted EdU solution for 2 h. Discard the base and wash it twice with PBS, 5 min each time. Add 50 *μ*L fixative solution to each well and incubate for 30 min at room temperature. After discarding the fixate, 50 *μ*L 2 mg/mL glycine was added to each well. Discard the glycine solution and wash it with PBS once. Incubate with 100 *μ*L penetrant decolorization shaker for 10 min in each well, and wash with PBS once. Add 50 *μ*L APOLLO staining solution to each well. Incubate in a decolorizing shaker at ambient temperature for 30 min. Add another 100 *μ*L of penetrant. Decoloring shaker cleaning is 2~3 times, each time 10 min. The osmotic agent was abandoned and cleaned 1-2 times with 100 *μ*L methanol per well and cleaned once with PBS. Finally, nuclear staining was photographed.

### 2.5. qRT-PCR

Total RNA was extracted according to the instructions of TRIzol reagent (Invitrogen, USA). The expression levels of target genes in CC cell lines were measured by SYBR Prime Script RTPCR Kit produced by Takara. The inverse system is as follows (20 *μ*l): SYBR Premix Ex TaqII (2×) 10 *μ*l, PCR Forward Primer (10 *μ*mol/L) 0.8 *μ*l, qPCR Primer (10 *μ*mol/L) 0.8 *μ*l, ROX Reference Dye II (50×) 0.4 *μ*l, cDNA template 2 *μ*l, and ddH_2_O 6 *μ*l. Reaction conditions are as follows: preconditioning at 95°C for 10 min; denaturation at 95°C for 10 s; annealing at 60°C for 10 s; and extension at 72°C for 15 s, 40 cycles. Primers were synthesized by Shanghai Sangon Bioengineering Company. The expression level of the target gene was expressed by the 2^-*△△*Ct^ method.

### 2.6. Transwell Experiment

48 hours after transfection, cells in each group were trypsinized to prepare single-cell suspension. After washing the cells twice with serum-free medium, cell counts were performed. Adjust the cell concentration to 1 × 10^5^/ml. 100 *μ*l of cell suspension was slowly dropped into the upper chamber. The small inner membrane material is polycarbonate (PC). The cell diameter is 8.0 *μ*m. 500 *μ*l of complete medium containing 10% fetal bovine serum was added to the lower chamber, and the cells were cultured at 37°C and 5% CO_2_-saturated humidity after planting. Wash the cells that have not penetrated the membrane in the upper chamber. Use a cotton swab to gently wipe away cells that have not penetrated the membrane. Fix with methanol for 15 min. Hematoxylin staining was performed for 10 min, and after air-drying, the polycarbonate membrane was gently cut with a razor blade. It was placed on glass slides and mounted with neutral gum. The number of cells passing through the membrane was observed under an upright fluorescence microscope. Five high-power fields of view were randomly selected and photographed and recorded.

### 2.7. Subcutaneous Tumor-Bearing Animal Model

Twelve male BALB/C nude mice, 4-5 weeks old, weighed (20 ± 2) g. They were raised in a specific pathogen-free (SPF) environment. Cells in logarithmic growth phase were trypsinized and centrifuged. Resuspend in serum-free RPMI 1640 medium to prepare a single-cell suspension with a cell density of 1 × 10^7^/L. Each 0.1 mL (about 2 × 10^6^ cells) was subcutaneously inoculated into the right forelimb of nude mice. After inoculation of SiHa cells, observation was performed once a day. The criteria for successful modeling were that the length and diameter of the tumor mass were greater than 5 mm, and the texture was hard to the touch. The nude mice were weighed, and the tumor volume was measured once every 2 days. The long diameter (*a*) and the vertical short diameter (*b*) of the transplanted tumor were measured with an electronic digital caliper each time. The tumor volume (TV) was calculated, TV = *a* × *b*^2^/2, and the growth curves of the transplanted tumors in each group were drawn. This study was approved by Animal Ethics Committee of Beijing Tongren Hospital. The animal experiments in this study strictly comply with laws, regulations, and standards related to experimental animals, including but not limited to regulations on the management of experimental animals and guidelines for ethical review of experimental animal welfare (GB/T 35892-2018). Animals were euthanized with carbon dioxide.

### 2.8. Immunohistochemistry

The mice were sacrificed, and the tumor mass was quickly removed and weighed. 4% paraformaldehyde was fixed, embedded in paraffin, and sectioned. Paraffin sections were routinely deparaffinized, antigen was retrieved, endogenous peroxidase was blocked, and primary antibodies were added, according to the kit instructions, DAB color development, different concentrations of ethanol dehydration, xylene transparent, and neutral resin seal. According to the detected primary antibody to the target protein, the intensity of brownish yellow is used as the observation index.

### 2.9. Western Blot Analysis

The cells 48 h after transient transfection were taken, and the medium was discarded. After washing 3 times with PBS, blot dry, add the prepared RIPA cell lysis buffer to fully lyse (1 × RIPA : 100 × CK : 100 × phosphatase inhibitor = 100 : 1 : 1). The lysate was sucked into the labeled EP tube, and it was shaken every 10 min on a vortex shaker for a total of 3 times. Centrifuge at 12000 × g for 30 min at 4°C and transfer the supernatant to a new EP tube. Protein concentration was determined with BCA protein quantification kit. Calculate the loading volume of 30 *μ*g sample according to the concentration of protein sample, and align the sample volume with lysis buffer. The loading buffer was prepared according to the volume ratio of protein sample to 5 × SDS loading buffer of 4 : 1. Boil in a 95°C water bath for 10 minutes. Configure 10% polyacrylamide gel, 80 V, 30 min; 100 V, 90 min electrophoresis; and 250 MA, 120 min transfer membrane. 5% nonfat milk powder was blocked for 60 min, and the primary antibody was added to incubate at 4°C overnight. Wash in TBST for 10 min × 3 times, add secondary antibody diluted in appropriate ratio, incubate at room temperature for 60 min, and wash in TBST for 10 min × 3. Expose and calculate the expression level of the target protein.

### 2.10. Statistical Analysis

All measurement data are expressed as mean ± standard deviation. The SPSS 16.0 statistical software was used for analysis. The comparison of sample means between the two groups was performed using Student's *t*-test. One-way ANOVA was used to compare multiple groups. *P* < 0.05 was considered to be statistically significant.

## 3. Results

### 3.1. In CC Patients, High Expression of SEC61G Predicts Poor Prognosis

**Figure 1:**
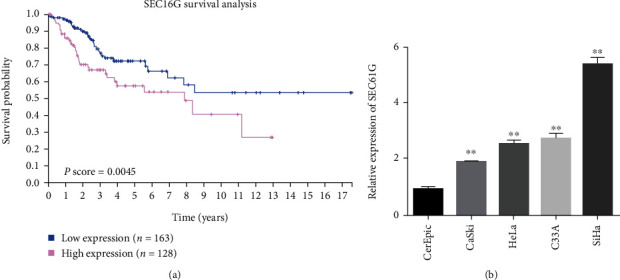
In CC patients, high SEC61G expression predicts poor prognosis. (a) Kaplan-Meier curve of overall survival in CC patients with high or low SEC61G expression levels in the cohort. The data sources of SEC61G survival analysis were downloaded from The Human Protein Atlas website (https://www.proteinatlas.org/). (b) SEC61G is highly expressed in CC cells. ^∗^*P* < 0.05 and ^∗∗^*P* < 0.01 vs. CerEpiC group.

Kaplan-Meier curves of overall survival in CC patients with high or low SEC61G expression levels were analyzed using TCGA database. The expression level of SEC61G is correlated with the prognosis of patients, and patients with high SEC61G expression have a short survival time (Figure 1(a)). Among the 4 CC cell lines, the expression level of SEC61G in SiHa cells was the highest, significantly higher than other cell lines (Figure 1(b)). Therefore, in subsequent experiments, we downregulated CC cell line SiHa with high SEC61G expression for further study.

### 3.2. Silencing SEC61G Inhibits Proliferation and Invasion of CC Cells In Vitro

**Figure 2:**
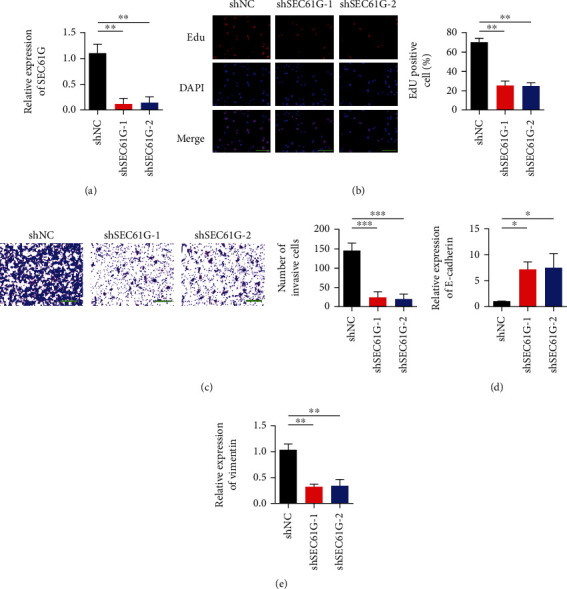
Silencing of SEC61G inhibits proliferation and invasion of CC cells in vitro. (a) The transfection efficiency of SEC61G was detected by transfecting SiHa (SiHa-shSEC61G) and control group (shNC) with SEC61G knockout shRNA. (b) EdU proliferation assay. (c) Transwell detection of invasive ability of CC cells. After 48 h, crystal violet staining was performed and counted. 200x. (d) Detection of E-cadherin expression. (e) Vimentin expression detection. ^∗^*P* < 0.05, ^∗∗^*P* < 0.01, and ^∗∗∗^*P* < 0.001 vs. shNC group.

The interference efficiency of SiHa cells was verified by qRT-PCR after transfection of sh-SEC61G and negative control. The results showed that the expression level of SEC61G was downregulated compared with the control group (Figure 2(a)). The changes of cell proliferation after SEC61G interference were detected by EdU experiment. The results showed that cell proliferation was weakened after interference with SEC61G (Figure 2(b)). As shown in Figure 2(c), after SEC61G was silenced, the number of transmembrane cells of SiHa cells decreased compared with the blank control group, and the difference was statistically significant (*P* < 0.01). After the interference efficiency was verified by transfection of sh-SEC61G and negative control in SiHa cells, the SiHa cells with instantaneous interference to SEC61G were taken and the changes of EMT marker expression were detected by qRT-PCR. Results showed that E-cadherin expression was upregulated, and Vimentin expression was downregulated after SEC61G downregulation (Figures 2(d) and 2(e)). These results suggest that SEC61G may regulate the EMT process in CC cells.

### 3.3. Overexpression of SEC61G Promoted Proliferation and Invasion of CC Cells In Vitro

**Figure 3:**
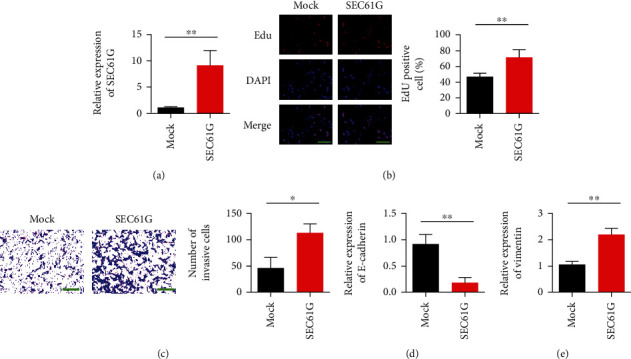
Overexpression of SEC61G promotes proliferation and invasion of CC cells in vitro. (a) SiHa cells were transfected with a plasmid overexpressing SEC61G, and the transfection efficiency of SEC61G was detected. (b) EdU proliferation assay. (c) Transwell detection of invasive ability of CC cells. After 48 h, crystal violet staining was performed and counted. (d) Detection of E-cadherin expression. (e) Vimentin expression detection. ^∗^*P* < 0.05 and ^∗∗^*P* < 0.01 vs. mock group.

After SiHa cells were transfected with mock and SEC61G overexpressed plasmids, the transfection efficiency was verified by qRT-PCR. The results showed that compared with the control group, the expression level of SEC61G was upregulated (Figure 3(a)). After overexpression of SEC61G gene in SiHa cells, EdU detected the effect of SEC61G on cell proliferation. The results showed that cell proliferation was enhanced after overexpression of SEC61G (Figure 3(b)). In addition, overexpression of SEC61G also enhanced the invasion ability of cells (Figure 3(c)). The expression of EMT markers was detected by qRT-PCR. The results showed that E-cadherin expression was decreased and Vimentin expression was upregulated after overexpression of SEC61G (Figures 3(d) and 3(e)).

### 3.4. SEC61G Promotes the Growth and Progression of CC In Vivo

**Figure 4:**
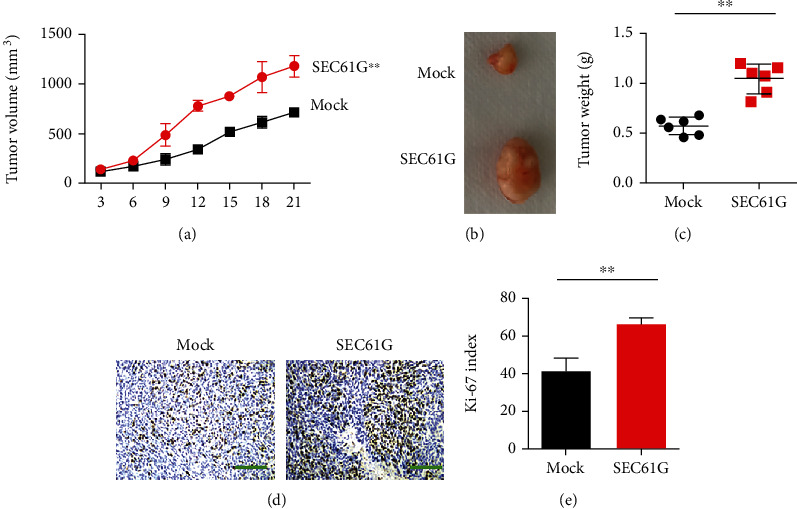
SEC61G promotes CC growth and progression in vivo. (a) Nude mice were subcutaneously implanted with SiHa cells overexpressing SEC61G (SiHa-SEC61G) and a control group (SiHa-mock). Tumor growth curves were measured at the indicated time points between SiHa-SEC61G and SiHa-mock. ^∗^*P* < 0.05, one-way ANOVA. (b) Each group was injected with 2 × 10^6^ cells; tumors were excised on day 21 postinjection. (c) Tumor weight was measured when the tumor was removed. (d) Representative images of IHC staining showing a positive correlation between Ki-67 and SEC61G overexpression in subcutaneous implants of each group. (e) Relative intensity of Ki-67 staining. ^∗∗^*P* < 0.01 vs. mock group.

SiHa cells were subcutaneously inoculated in the right forelimb of nude mice. On day 7, hard tissue could be seen in the inoculated site and reached the modeling standard. The modeling success rate was 100%. The transplanted tumor growth curve was made with time as abscissa and graft volume as ordinate. After overexpression of SEC61G, the growth rate of transplanted tumors was faster than that of the negative control group (Figures 4(a) and 4(b)). The influence of SEC61G on tumor weight is shown in Figure 4(c). After overexpression of SEC61G, tumor weight of CC increased. Representative images of IHC staining showed a positive correlation between Ki-67 and SEC61G overexpression in subcutaneous implants in each group (Figures 4(d) and 4(e)).

### 3.5. Knocking SEC61G Inhibits MAPK Signaling Pathway

**Figure 5:**
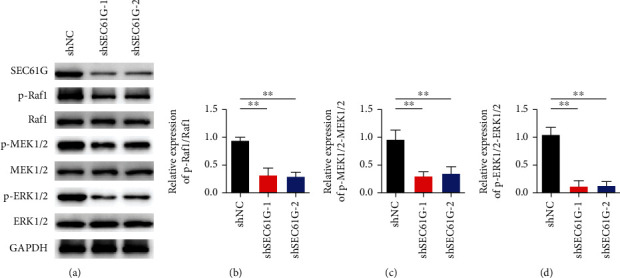
Knockdown of SEC61G inhibits MAPK signaling. (a) Western blot detection of key protein expression changes of MAPK signaling pathway. (b) Statistical analysis results of p-RAF1/Raf1. (c) Statistical analysis results of p-MEK1/2-MEK1/2. (d) Statistical analysis results of p-ERK1/2-ERK1/2. ^∗∗^*P* < 0.01 vs. shNC group.

As shown in Figure 5(a), compared with the blank control group, changes in key protein expression levels of MAPK signaling pathway were detected by Western blot after SEC61G was silenced. The results showed that p-RAF1/Raf1, p-MEK1/2-MEK1/2, and p-ERK1/2-ERK1/2 expression levels were all decreased after SEC61G was knocked out (Figures 5(b)–5(d)).

### 3.6. Gefitinib Can Inhibit CC Proliferation and Tumor Growth by SEC61G

**Figure 6:**
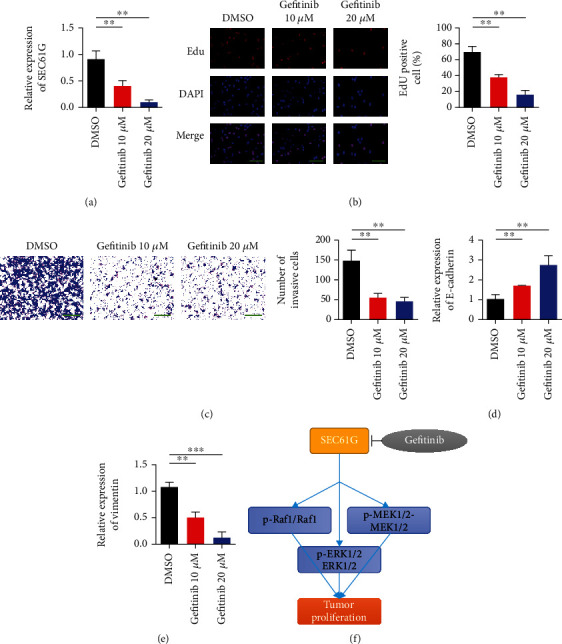
Gefitinib inhibits CC proliferation and tumor growth through SEC61G. (a) After SiHa cells were given gefitinib (10 *μ*M, 24 h) or DMSO, the expression of SEC61G was analyzed by qRT-PCR. (b) EdU proliferation assay. (c) Transwell detection of invasive ability of CC cells. After 48 h, crystal violet staining was performed and counted. (d) Detection of E-cadherin expression e. (e) Vimentin expression detection. (f) Schematic description of the regulatory mechanism of CC proliferation through SEC61G/MAPK signaling and therapeutic targets. ^∗∗^*P* < 0.01 and ^∗∗∗^*P* < 0.001 vs. DMSO group.

Based on our understanding of SEC61G, we verified the inhibitory effect of chemotherapy drug gefitinib on SEC61G. SiHa cells were treated with gefitinib (10 *μ*M, 24 h) or DMSO, and the expression level of SEC61G was analyzed by qRT-PCR. The results showed that the expression of SEC61G was significantly inhibited after gefitinib was administered to SiHa cells (Figure 6(a)). EdU and Transwell results showed that gefitinib reduced proliferation and invasion ability of CC cells by inhibiting SEC61G (Figures 6(b) and 6(c)). Changes in EMT marker expression showed that E-cadherin expression was upregulated after gefitinib treatment, while Vimentin expression was upregulated (Figures 6(d) and 6(e)).  Figure 6(f) shows the regulation mechanism of SEC61G/MAPK signal axis on CC proliferation.

## 4. Discussion

Cervical cancer (CC) is one of the common malignant tumors [14]. The primary site of the tumor is hidden, difficult to detect early, with high metastasis, high recurrence, and low differentiation and other characteristic [15]. The occurrence of CC is a complex biological process involving multiple factors and stages. It involves the abnormal activation of multiple signal transduction pathways and the abnormal activation of multiple protein kinases, but the specific mechanism remains to be further elucidated [16, 17].

We interfered with the SEC61G gene in CC cell line SiHa. Compared with the control group, cell proliferation was reduced by cell proliferation assay. These results suggest that SEC61G can promote the proliferation of CC cell line SiHa. Western blot analysis showed that downregulation of SEC61G could weaken the expression of key proteins in MAPK signaling pathway. These results suggest that SEC61G may promote the proliferation of CC cell SiHa by promoting MAPK signaling pathway. These results suggest that SEC61G may be an important oncogenic factor in the development and progression of CC and may provide a new direction for the diagnosis and treatment of CC.

There are five MAPK signaling pathways in mammals, which play a comprehensive role in tumor proliferation, apoptosis, invasion, and metastasis, and play an important role in the occurrence and development of tumors [18]. MAPK signaling pathway can be activated by a variety of factors, and its ways of action are complex and diverse [19]. Its upstream kinase and downstream species are complex, and there are many influencing factors [20]. The biological effects of activation are varied. Some stimuli can promote the activation of MAPK/ERK pathway, and the activated p-ERK on the one hand transfers into the nucleus and induces the successive activation of various oncogenes through transcriptional regulation, resulting in malignant transformation, abnormal proliferation, and tumor generation [21].

ERK1/2 is the earliest mammalian pathway that plays an important role in tumor cell proliferation [22]. It can be activated by ligands of growth factors, proteases, polysaccharides, g-protein-coupled receptors, etc. The ERK signal transduction pathway is triggered primarily by signals transmitted by Ras or protein kinase C [23]. Raf is then activated to initiate the cascade reaction between mitogen-activated protein kinase kinase (MEK) and ERK activation [24]. In this study, it was found that p-RAF1/Raf1, p-MEK1/2-MEK1/2, and p-ERK1/2-ERK1/2 activities were increased after overexpression of SEC61G, which promoted the growth and proliferation of CC cells. ERK/MAPK signal transduction pathway is related to the expression pathways of many factors. Blocking ERK/MAPK signal transduction pathway can affect the expression of many factors and prevent the formation of tumor blood vessels to a certain extent, thus inhibiting the development of tumor. In this study, inhibition of SEC61G with gefitinib was found to reduce the viability and proliferation of cancer cells.

## 5. Conclusion

In conclusion, this study confirmed that SEC61G plays an important role in CC cell proliferation and transformation. The mechanism may be related to its regulation of MAPK signaling pathway. Inhibition of SEC61G expression or activity is expected to be a new strategy for tumor therapy.

## Data Availability

The data used to support the findings of this study are available from the corresponding author upon request.
